# Low expression of the X-linked ribosomal protein S4 in human serous epithelial ovarian cancer is associated with a poor prognosis

**DOI:** 10.1186/1471-2407-13-303

**Published:** 2013-06-22

**Authors:** Serges P Tsofack, Liliane Meunier, Lilia Sanchez, Jason Madore, Diane Provencher, Anne-Marie Mes-Masson, Michel Lebel

**Affiliations:** 1 Centre de Recherche en Cancérologie de l’Université Laval, Hôpital Hôtel-Dieu de Québec, Quebec City, QC, Canada; 2 Institut du cancer de Montréal, Montréal, QC, Canada; 3 Centre de recherche du Centre hospitalier de l’Université de Montréal (CRCHUM), Montreal, QC, Canada; 4 Centre de Recherche en Cancérologie de l’Université Laval, Hôpital Hôtel-Dieu de Québec, 9 McMahon Sreet, Quebec City, QC G1R 2J6, Canada

**Keywords:** Serous epithelial ovarian cancer, YB-1, RPS4X, Cisplatin

## Abstract

**Background:**

The X-linked ribosomal protein S4 (RPS4X), which is involved in cellular translation and proliferation, has previously been identified as a partner of the overexpressed multifunctional protein YB-1 in several breast cancer cells. Depletion of RPS4X results in consistent resistance to cisplatin in such cell lines.

**Methods:**

As platinum-based chemotherapy is a standard first line therapy used to treat patients with ovarian cancer, we evaluated the prognostic value of RPS4X and YB-1 at the protein level in specimen from 192 high-grade serous epithelial ovarian cancer patients.

**Results:**

Immunohistochemistry studies indicated that high expression of RPS4X was associated with a lower risk of death and later disease progression (HR = 0.713, *P* = 0.001 and HR = 0.761, *P* = 0.001, respectively) as compared to low expression of RPS4X. In contrast, YB-1 was not significantly associated with either recurrence or survival time in this cohort. Finally, the depletion of RPS4X with different siRNAs in two different ovarian cancer cell lines reduced their proliferative growth rate but more importantly increased their resistance to cisplatin.

**Conclusion:**

Altogether, these results suggest that the levels of RPS4X could be a good indicator for resistance to platinum-based therapy and a prognostic marker for ovarian cancer. Our study also showed that RPS4X is an independent prognostic factor in patients with serous epithelial ovarian cancer.

## Background

Epithelial ovarian cancer (EOC) is a disease responsible for more cancer deaths among women in the Western world than all other gynecologic malignancies. Because of its asymptomatic nature, ovarian cancer is characterized at presentation with advanced disease having spread primarily via an intraperitoneal route. An initial surgical approach is essential for proper staging of the disease process and for aggressive cytoreduction, which in turn improves the response to chemotherapy and survival [1]. Chemotherapy has had an increasingly important role in the effective treatment of ovarian cancer. The reference standard for postsurgical ovarian cancer first-line chemotherapy has been the use of a platinum–taxane combination [2]. Although the standard platinum-taxane regimen results in a response rate of 80% in advanced ovarian cancer patients, most of these patients relapse after a median period of 18 months, due to the emergence of tumor resistance to these conventional drugs [3-5]. Thus, there is an immediate need for the identification of pharmacogenomic markers to identify patients unlikely to respond, those that will relapse rapidly, or patients at risk for severe toxicity.

In recent years, several studies have reported the involvement of YB-1 in patient survival and cisplatin resistance in ovarian cancers [6,7]. The YB-1 protein is a multifunctional protein that affects the transcription, splicing, and translation of specific mRNAs [8-11]. Increased expression of YB-1 is associated with a poor prognosis in ovarian cancer [7]. YB-1 binds preferentially to cisplatin-modified DNA [12] and interacts with several DNA repair proteins [13,14]. Although YB-1 affects several biological processes, it is still unknown which ones are important for cisplatin resistance. In a recent study of breast cancer cell lines, we identified the proteins that interact directly to YB-1 and impact on cisplatin response upon depletion [15]. Interestingly, we found that the small ribosomal protein 4X (RPS4X) increases cisplatin resistance upon depletion with specific small interference RNAs. As platinum-based compounds are used in the treatment of ovarian cancers, we sought to correlate the levels of RPS4X in clinical samples with patient survival and disease progression.

In this work, we determined by immunohistochemistry the levels of both RPS4X and YB-1 in ovarian cancer samples from patients who were treated with a platinum-based chemotherapeutic regimen after their surgery. RPS4X not only correlated with stage, but low levels of RPS4X also correlated with poor survival and disease progression. These results indicate that RPS4X could be a predictive and prognostic marker in ovarian cancer.

## Methods

### Ethics statement

Ethics approval for specimen collection and the study were obtained by the local institutional ethics board (Comité d’éthique de la recherche du Centre hospitalier de l’Université de Montréal).

### Patients and tissue specimens

Tumor samples were collected and banked following appropriate consent from patients undergoing surgery within the Division of Gynecologic Oncology at the Centre hospitalier de l’Université de Montréal from 1993 to 2010. An independent dedicated GYN-pathologist scored the tumor grade and subtype and a gynecologic oncologist scored the stage and the tumor residual disease according to criteria from the International Federation of Gynecologists and Obstetricians [16]. Clinical data on progression-free interval were defined according to RECIST 1.1 [17]. Overall survival was defined as the time from surgery to death from ovarian cancer. Patients known to be still alive at time of analysis were censored at time of their last follow-up. Patient disease free survival (DFS) was calculated from the time of surgery until the first progression. Eligibility criteria for inclusion in the study were as follows: primary surgery, complete information on post-operative chemotherapeutic treatment, high grade serous histopathology subtype, and completed tumor banking informed consent. Patients who died from another disease were censored at time of last follow-up. A gynecologic oncologist reviewed the clinical data for all patients. For the disease-free progression study, only patients with clinical follow-up of at least 18 months or until disease recurrence were included. The characteristics of the tumors and patient outcome for the sample sets are summarized in Table 1.

**Table 1:** Description of the high-grade serous ovarian carcinomas (HGSOC) tissue array

**Variable**	**N = 192**
	n (%)
Stage	
I	10 (5.2)
II	21 (10.9)
III	135 (70.3)
IV	26 (13.5)
Res. Disease	
Negative	26 (13.5)
Milliary	5 (2.6)
<1 cm	29 (15.1)
1-2 cm	16 (8.3)
2 cm	63 (32.8)
Variable	N = 192
	mean (SD)
Age, years	62 (11)
Disease free survival, month	22 (26)
Overall survival, month	35 (29)

Stage is divided in 4 categories according to FIGO classification. Residual disease at surgery time (Res. Disease) was evaluated by a gyneco-oncologist (in cm). negative = no residual disease. milliary = very small, discrete, multiple areas of tumor tissue, and is grouped by convention with the <1 cm group. Abbreviations: *SD* standard deviation.

### Tissue microarray (TMA)

Areas of tumor were selected based on review of a hematoxylin-eosin-stained slide. All samples were fixed with formalin and embedded in paraffin following a standard procedure. Formalin fixed paraffin embedded tumor blocks were then biopsied using a 0.6 mm diameter tissue arrayer and resultant cores were arrayed into a grid in a recipient paraffin block. It has previously been demonstrated using several different antibodies that the quality of the core samples on this TMA was suitable for immunohistochemistry and statistical analyses confirmed that the age of the paraffin blocks was not a confounder in these studies [18]. The tissue array was composed of 260 ovarian cancer samples from patients that never received chemotherapy before their surgery and 11 samples of areas from normal fallopian tubes of cancer patients. After review of the clinical data 68 patients were excluded from the final analysis, as they did not meet the study inclusion criteria. For the RPS4X immunostaining study, two core samples on the TMA were damaged and thus excluded (thus N = 190). For the YB-1 immunostaining study, six core samples were excluded for similar reason (thus N = 186). The completed tissue array was sectioned, stained with hematoxylin-eosin and received another pathology review to confirm tumor content [18].

### Immunohistochemistry

The TMA of formalin fixed paraffin embedded tumors was sectioned at 4 μm and slides were stained using the BenchMark XT automated stainer (Ventana Medical System Inc.). The optimal concentration for each primary antibody was determined by serial dilutions. The rabbit polyclonal antibody against human RPS4X (14799-1-AP) was purchased from ProteinTech Group, Inc. (Chicago IL). A polyclonal antibody against the N-terminus portion of YB-1 (ab12148) was purchased from Abcam, Inc. (Cambridge, MA) [19,20]. The rabbit monoclonal antibody against Ki67 (RM-9106) was purchased from Lab Vision (Fremont, CA). Nuclei were counterstained with hematoxylin. Antigen retrieval was carried out with Cell Conditioning 1 (Ventana Medical System Inc.; #950–124) for 30 min (YB-1 and RPS4X) or 60 min (Ki67). Pre-diluted antibody was automatically dispensed, and the slides were incubated at 37°C for 60 min (YB-1 and RPS4X) or 44 min (Ki67). Reactions were carried out using the UltraView DAB detection kit (Ventana Medical System Inc.; #760–091). Slides were counterstained with hematoxylin (Ventana Medical System Inc.; #760–2021). All sections were scanned with a 20x 0.75NA objective with a resolution of 0.3225 μm. Substitution of the primary antibody with phosphate buffered saline served as a negative control.

### Staining quantification

Tumor sections were scanned, digitally conserved, and manually visualized. For RPS4X and YB-1, a score was given to each core according to the staining intensity of the cytoplasm in the epithelial cells from 1 (weak) to 5 (strong). For both markers, no cores presented negative staining. For Ki67, cores were scored for the percentage (rounded to the nearest 5%) of total staining. Each array was independently analyzed in a blind study by two independent observers. We use the inter-rating correlation (Cronbach’s Alpha) to evaluate the overall correlation between the observers as described previously [18]. Inter-rating correlation was >75% for all three proteins. The average score from the two independent observers, for each respective core, was used for analysis.

### YB-1 and RPS4X knock down

The human OVCAR-3 and SK-OV-3 serous ovarian cancer cell lines were obtained from the American Type Culture Collection (ATCC). The OVCAR-3 cells were maintained in RPMI media supplemented 15% Fetal Bovine Serum (FBS) and 1% Penicillin-Streptomycin (Invitrogen, Carlsbad, CA) at 37°C in atmosphere of 5% CO_2_. The SK-OV-3 cells were maintained in DMEM supplemented with 10% FBS and 1% Penicillin-Streptomycin. To deplete RPS4X or YB-1 proteins in cells, small interference RNA (siRNA) molecules were transfected with the Lipofectamine 2000 as described by the manufacturer (Invitrogen, Carlsbad, CA). The knock down efficiency was confirmed by western blot analyses with antibodies against YB-1, RPS4X, and β-actin as control. Horseradish peroxidase-conjugated secondary antibodies (anti-rabbit IgG: NAV934V and anti-mouse IgG: NA931V) were purchased from GE Healthcare Limited (Piscataway, NJ). The siRNA sequences against YB-1 are 5′-AAGAAGAAAUAUGAAAUUCCA-3′ for the siRNA-A molecule and 5′-CUGCAAGCACCUGUUAAUAAA-3′ for siRNA-B. The siRNA sequences against RPS4X are 5′-CAGAUCUUUGUACGUAAUUAA-3′ for the siRPS4X-A molecule and 5′-CGGGAGAGAAUUUCCGUCUGA-3′ for siRPS4X-D. A scrambled control siRNA was purchased from Invitrogen (Carlsbad, CA).

To obtain the growth curves of transfected cells, 10,000 OVCAR-3 or 50,000 SK-OV-3 transfected cells were plated in 60 mm dishes and counted with a hemacytometer by the trypan blue exclusion technique every other day. Experiments were performed in triplicate.

### Western blots

All transfected and untransfected cells were lysed in RIPA buffer [50 mM Tris–HCl (pH 7.5), 150 mM NaCl, 1% NP-40, 0.1% SDS, 0.5% sodium deoxycholate] for SDS-PAGE analyses. Proteins from SDS-PAGE were transferred onto Amersham Hybond-P membranes (GE Healthcare Limited, Piscataway, NJ). Membranes were blocked one hour at room temperature in PBS containing 5% milk/0.1% Tween, washed in PBS-Tween (0.1%), and incubated overnight with the primary antibodies in PBS containing 5% milk overnight at 4°C. Blots were washed the next day in PBS-Tween and incubated two hours at room temperature with horseradish peroxidase-conjugated secondary antibody in PBS containing 5% milk. Blots were washed with PBS-Tween and proteins were revealed with chemiluminescence reagents (ECL Plus; GE Healthcare Limited, Piscataway, NJ). Immunoprecipitation of GFP (Green Fluorescent Protein) and GFP-YB-1 constructs were performed as described previously [21]. Protein bands on western blots were quantified using LI-COR Image Studio software 2.0 (LI-COR Biosciences, Lincoln, NE). β-actin was used as a control for protein loading. The background signal for each band was determined using an identical area to the target band covering a region in the same lane where no protein signal was observed. Results were determined by calculating a ratio of target protein signal (minus background) over β-actin signal (minus background).

### Immunofluorescence analysis

SK-OV-3 cells were plated on coverslips and transfected the next day with control siRNA or siRNA sequences against RPS4Xm RNA. Three days later cells were fixed in 4% paraformaldehyde for 20 min at room temperature (RT) and permeabilized with 0.15% Triton X-100 at RT for 10 min. After washing with PBS, cells were blocked with 3% BSA at room temperature for 30 min. After blocking, the antibody against RPS4X was diluted in 1% blocking buffer (1:100) and applied to the coverslips for an overnight incubation at 4°C. The next day, coverslips were washed with PBS and incubated with rhodamine-secondary antibody (Santa Cruz) for 1 h30 min in the dark at RT. After washing, coverslips were stained with DAPI 10 min, washed, and mounted on glass slides. Slides were viewed at 60X magnification (1.4NA oil-immersion 60X objective) and zoomed 2X for image acquisition on a Nikon inverted diaphot confocal microscope equipped with Krypton/Argon lasers (488 and 568 nm). Images were captured with a BioRad MRC1024 confocal microscopy system. Finally, images were analyzed (colored and merge) using the Fiji-win32 software.

### FACS and FITC-Annexin V analyses

Cells were transfected with either control siRNA or siRNA against RPS4X. After 72 h, cells were fixed in 50% ethanol overnight. Cells were then washed in phosphate-buffered saline (PBS) and incubated for 30 min at 37°C in a buffer containing propidium iodide and RNAses. Cells were then analyzed on a Beckman-Coulter Epics Elite ESP (Cambridge, MA, USA) flow activated cell sorter. Data were analyzed with the MultiCycle software (Phoenix Flow System, San Diego, CA, USA). To estimate apoptosis and/or necrosis, we used the FITC Annexin V apoptosis detection kit I (BD Biosiences, Palo Alto, CA). Transfected cells were treated 48 h with the indicated concentration of cisplatin and then harvested to measure apoptosis/necrosis following the manufacturer’s instructions.

### Cisplatin treatment and sulforhodamine B colorimetric assay

Cells were transfected with the indicated siRNAs and allowed to grow for 24 hours. The next day, 10,000 cells were seeded per well on a 96-well plate and incubated at 37°C for 24 hours. Different concentrations (0–40 μM) of cisplatin were added to the cells in triplicate and cells were then allowed to grow for an additional 48 hours. Cells were fixed with tricholoroacetic acid (10% w/v) and stained 30 min with sulforhodamine B as described [22].

### Statistical analysis

The Spearman correlation (two-tailed) and non-parametric Wilcoxon-Mann–Whitney test were used to estimate the correlation with clinicopathological variables and markers as continuous variables. Survival curves were calculated according to Kaplan-Meier method coupled with a log-rank test for survival analysis. Since survival times were positively skewed, we took the median as the threshold value for each marker (YB-1 and RPS4X). Univariable and multivariable Cox proportional hazard models were used to estimate the hazard ratio for each marker as continuous variables. All statistical analyses were done using Statistical Package for the Social Sciences software version 16.0 (SPSS, Inc.), and statistical significance was set at *P* < 0.05.

The R software version 2.10.1 (http://www.r-project.org/) was used to estimate the growth rate, the IC50, and the associated standard deviation. Briefly, the growth curves were fitted to a mathematical model of the form y = x_0_*(1 + r)^t^, where x_0_ represents the 50,000 transfected cells plated on day 0, r represents the growth rate, and t represents the time unit (days). The dose response curves were fitted to a standard exponential decay mathematical model of the form y = y_0_ + A*e^kx^ where y_0_ represents the minimal normalized intensity, A the intensity at time 0 and k is the decay rate.

## Results

### RPS4X and YB-1 expression in ovarian cancer samples

**Figure 1:**
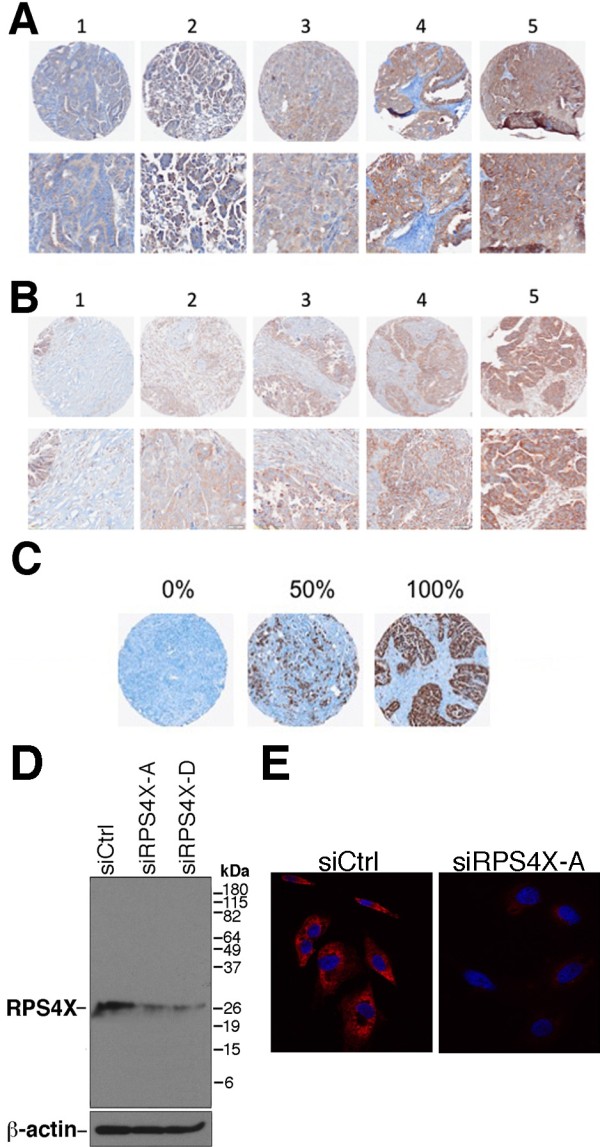
**Representative staining for immunohistochemistry of YB-1, RPS4X, and Ki67 on a high-grade serous EOC TMA. A**, representative staining of each intensity by immunohistochemistry for YB-1. From left to right: low to high intensity. **B**, representative staining of each intensity by immunohistochemistry for RPS4X. From left to right: low to high intensity. **C**, representative staining of each intensity by immunohistochemistry for Ki67. From left to right: 0% of total staining, 50% of total staining, and 100% of total staining. **D**, western blot analysis of total protein extracts from SK-OV-3 cell lines transfected with a siRNA sequence against RPS4X mRNA (siRPS4X-A and siRPS4X-D) or transfected with a control (scrambled) siRNA sequence. β-actin is used as a loading control. **E**, a representation of immunofluorescence signals in SK-OV-3 cells transfected with a siRNA sequence against RPS4X mRNA (siRPS4X-A) or transfected with a control scrambled siRNA sequence (siCtrl). Nuclei are revealed by DAPI staining (in blue).

It has been reported that YB-1 is overexpressed in ovarian cancers [7,23]. Since we recently found an interaction between RPS4X and YB-1 proteins [15] that may affect clinical outcomes of patients, we determined the levels of expression of these proteins by immunohistochemistry in 192 clinical samples from women with ovarian cancer (Table 1). (Note that for each immunohistochemistry staining experiments, samples of poor quality were excluded from the statistical analyses). We correlated the expression of these proteins with the mitotic index marker Ki67 from the same samples. Figure 1 demonstrates examples of the staining pattern obtained with the antibodies used against YB-1, RPS4X, and Ki67. Expression of proteins in the epithelium of the ovarian cancer tissues was observed and scored according to the intensity of staining as low to strong (1 to 5) (Figure 1). Both YB-1 and RPS4X were mainly cytoplasmic, while Ki67 gave a nuclear staining. The quality and validity of the antibodies against YB-1 and Ki67 used in this immunohistochemistry study have been described previously [18,19]. To confirm the validity of the antibody against RPS4X, we performed western blot and immunofluorescence tests on control and RPS4X-depleted SK-OV-3 ovarian tumor cell lines. As indicated in Figure 1D, the RPS4X antibody recognized a band of approximately 29 kDa that was depleted by two different siRNAs specific to RPS4X mRNAs. The immunofluorescence signal was also reduced in a population of SK-OV-3 cells transfected with a siRNA against RPS4X compared to siRNA control cells (Figure 1E). These results indicate that the antibody is specific to the RPS4X protein.

We investigated whether YB-1 was associated with RPS4X and Ki67 expression in ovarian cancer. Overexpression of YB-1 correlated significantly with total expression of Ki67 and expression of RPS4X in our clinical samples (Table 2). As expected, the expression of RPS4X correlated significantly with the expression of YB-1. It also correlated positively with the expression of the mitotic index marker Ki67.

**Table 2:** Spearman correlation test (two-tailed) for YB-1 and PRS4X expression (intensity) in EOC tissues and clinical data of patients

		**YB-1**	**RPS4X**	**Ki67 % total**	**Stage**	**Age**	**Residual disease**
YB-1	correlation	1	0.391**	0.315**	−0.016	0.004	−0.053
	Sig. (2-tailed)		<0.001	<0.001	0.829	0.961	0.535
	N	186	186	180	186	186	137
RPS4X	correlation	0.391**	1	0.224**	−0.180*	0.041	−0.193*
	Sig. (2-tailed)	<0.001		0.002	0.013	0.557	0.022
	N	186	190	183	190	190	141

* Correlation is significant at the 0.05 level (2-tailed).

** Correlation is significant at the 0.01 level (2-tailed).

Spearman correlation test were performed on clinical data, total Ki67%, YB-1, and PRS4X staining intensity observed in the intra-epithelial area of ovarian tumors. Stage was evaluated according to the FIGO classification. “Sig.” in the table represents the P-value from Spearman correlation test. N is the number of cases included in the statistical analysis.

**Figure 2:**
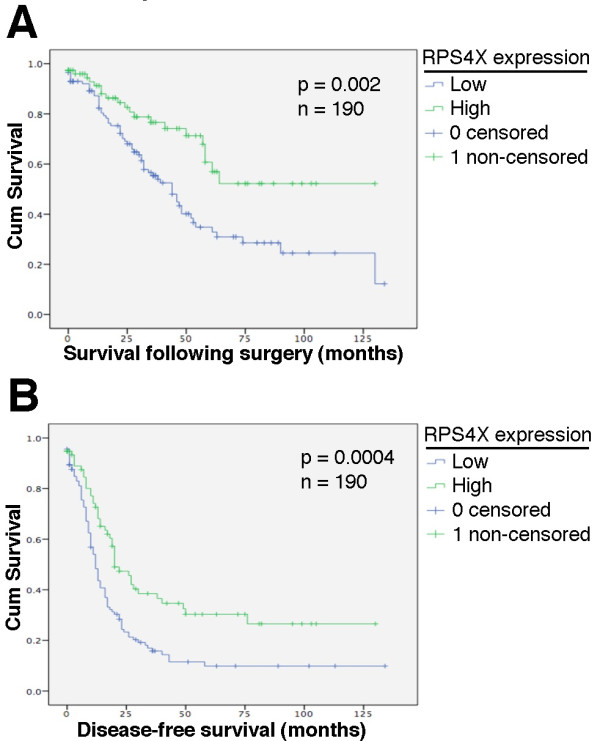
**Kaplan**-**Meier survival for low and high intensity of RPS4X in high grade serous EOC. A**, Kaplan-Meier curves of survival in our cohort. **B**, Kaplan-Meier curves of disease-free survival in our cohort. N = number of patients. Significance (p) is indicated by log-rank test. Subjects with low or high expression of RPS4X are plotted (low = scores 1 to 3; high = scores 4 to 5 from Figure 1B).

We next investigated the correlation between clinicopathological features of ovarian cancer cases and the expression of YB-1 and RPS4X. We determined whether the expression of YB-1 and RPS4X were associated with survival time and disease recurrence in patients with ovarian cancer using Kaplan-Meier plots. YB-1 was not significantly associated with either survival or recurrence time in our cohort (see Additional file 1: Figure S1). In contrast, Kaplan-Meier plots for RPS4X showed that the high expression of this biomarker is strongly associated with an increased overall patient survival (*P* = 0.002) (Figure 2A). Progression time was also significantly shorter in patients with low RPS4X expression (*P* = 0.0004) (Figure 2B). RPS4X also correlated significantly with lower levels of residual disease (Table 2) and with a lower disease stage (Table 2 and see Additional file 2: Table S2). Finally, YB-1 and RPS4X expression levels did not significantly correlate with patient age at diagnosis (Table 2).

In univariable Cox regression analysis, the level of RPS4X protein was evaluated to reflect the relation between decreasing levels of RPS4X expression and adverse prognosis. In this analysis, high expression of RPS4X is associated with a high hazard risk (HR) for survival (HR = 0.713; 95% Confidence Interval [CI] = 0.583-0.873, *P* = 0.001) (Table 3). It was also observed that higher RPS4X expression was associated with a longer time to disease progression (HR = 0.761; 95%CI = 0.652-0.888, *P* = 0.001). In multivariable Cox regression analysis, when standard prognostic variables were considered (age, stage and residual disease), RPS4X remained an independent variable predicting a high risk of survival (HR = 0.689; 95%CI = 0.545-0.871, *P* = 0.002) and a late risk of progression in the multivariable model (HR = 0.751; 95%CI = 0.626-0.901, *P* = 0.002) (Table 3).

**Table 3:** Cox regression analyses representing the statistical association between RPS4X expression and outcome in patients with high-grade serous ovarian cancer patients

		**Univariable analysis**			**Multivariable analysis**		
		**HR**	**(95%CI)**	**P**	**HR**	**(95%CI)**	**P**
Survival	age	0.998	(0.977 1.019)	0.841	0.999	(0.974 1.025)	0.952
	stage	1.713	(1.179 2.490)	0.005	1.432	(0.804 2.552)	0.223
	Res. Dis.	1.926	(1.490 2.491)	0.000	1.732	(1.309 2.290)	0.000
	RPS4X	0.713	(0.583 0.873)	0.001	0.689	(0.545 0.871)	0.002
Progression	age	0.993	(0.976 1.009)	0.381	0.984	(0.965 1.004)	0.110
	stage	2.238	(1.684 2.973)	0.000	1.877	(1.257 2.805)	0.002
	Res. Dis.	1.714	(1.425 2.060)	0.000	1.552	(1.262 1.908)	0.000
	RPS4X	0.761	(0.652 0.888)	0.001	0.751	(0.626 0.901)	0.002

Res. Dis. = amount of residual disease at time of primary resection of ovarian tumor. Age = age of patient at the diagnosis. HR = hazard ratio. CI = confidence interval. P = p value.

To summarize, all our statistical analyses indicate that high expression of RPS4X is associated with less aggressive ovarian tumors, slower disease progression, and with less deaths associated with this disease.

### Impact of RPS4X depletion on the growth of two serous epithelial ovarian cancer cell lines

**Figure 3:**
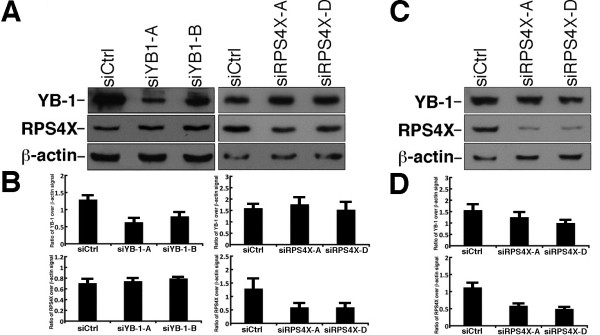
**Western blot analysis of total protein extracts from OVCAR-3 and SK-OV-3 cell lines transfected with different siRNA sequences. A**, OVCAR-3 cells were transfected with the indicated siRNA molecule and 48 hours later total protein extracts were purified, loaded on SDS/PAGE gel, and membranes were hybridized with anti-YB-1, anti-RPS4X, and anti-β-actin. Representative blots are presented. **B**, Histograms presenting the ratio of YB-1 or RPS4X signal over β-actin signal from the western blots in panel A. **C**, SK-OV-3 cells were transfected with the indicated siRNA molecule and 48 hours later total protein extracts were purified, loaded on SDS/PAGE gel, and membranes were hybridized with anti-YB-1, anti-RPS4X, and anti-β-actin. Representative blots are presented. **D**, Histograms presenting the ratio of YB-1 or RPS4X signal over β-actin signal from the western blots in panel C. All experiments were performed in duplicate.

We examined the effect of depleting YB-1 protein on RPS4X levels in the ovarian tumor line OVCAR-3. As indicated in Figure 3, a depletion of YB-1 protein (by approximately two-fold) with two different siRNAs did not have a significant effect on RPS4X protein levels (Figure 3A and B). Two different siRNAs against our target proteins were used in all experiments to avoid confounding results due to potential off target effect of a single siRNA [15]. Similarly, a two-fold depletion of RPS4X protein did not have a significant effect on YB-1 protein levels (Figure 3B). These results suggest that RPS4X and YB-1 do not regulate each other at the protein expression level in OVCAR-3 cells. The depletion of RPS4X in SK-OV-3 cells with the siRPS4X-D sequence decreased YB-1 protein levels by 33% only (Figure 3C and D). In contrast, the siRPS4X-A sequence did not decrease YB-1 protein level significantly (less than 14%) compared to the siControl transfection based on the overlap of the error bars of the histogram in Figure 3D.

**Figure 4:**
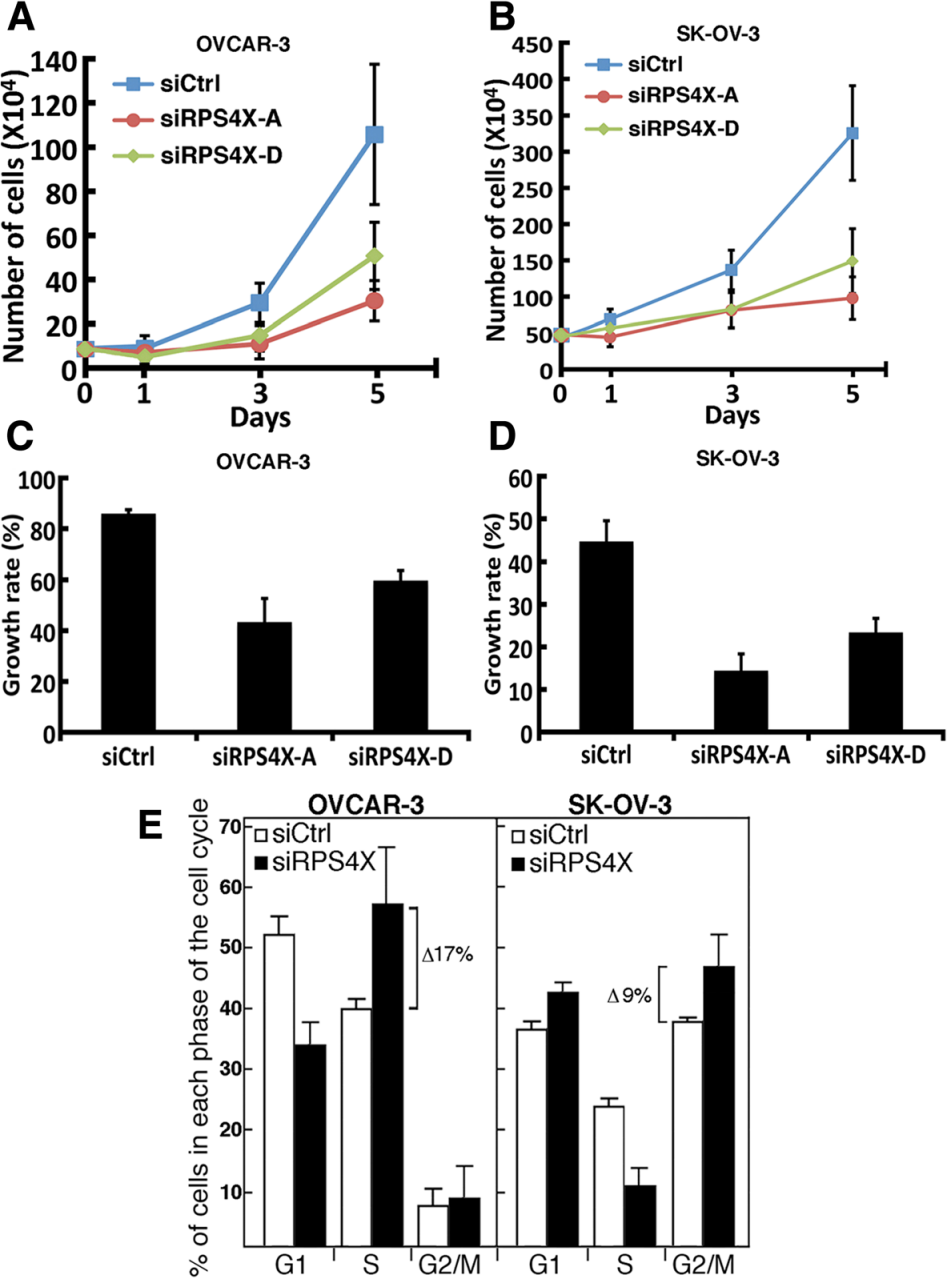
**Cell growth in RPS4X-depleted OVCAR-3 and SK-OV-3 cells. A**, cell growth of OVCAR-3 cells transfected with siCtrl, siRPS4X-A and siRPS4X-D molecules. Cells were transfected with the indicated siRNA sequences. The next day 10,000 cells were seeded in 60-mm plates and appropriate plates were counted every other day using a hemacytometer. **B**, Cell growth of SK-OV-3 cells transfected with siCtrl, siRPS4X-A and siRPS4X-D molecules. Cells were transfected with the indicated siRNA sequences. The next day 50,000 cells were seeded in 60-mm plates and appropriate plates counted every other day using a hemacytometer. **C**, Histogram representing the growth rate of OVCAR-3 transfected cells (from at least three transfections for each siRNA sequence) calculated from the growth curves in A. Error bars represent the standard deviation. (Unpaired student *t*-test: *P* = 7.6 × 10^-5^ for siRPS4X-A *vs* siCTRL and *P* =2.4 × 10^-6^ for siRPS4X-D *vs* siCTRL). **D**, Histogram representing the growth rate of SK-OV-3 transfected cells (from at least three transfections for each siRNA sequences) calculated from the growth curves in B. Error bars represent the standard deviation (Unpaired student *t*-test: *P* = 3.9 × 10^-7^ for siRPS4X-A *vs* siCTRL and *P* = 8.7 × 10^-6^ for siRPS4X-D *vs* siCTRL). Growth rates were estimated as described in materials and methods. **E**, Percentage of OVCAR-3 and SK-OV-3 transfected cells in each phase of the cell cycle. Cells were transfected with the indicated constructs in duplicates and subjected to FACS analysis 72 h later. (The siRPS4X represent data from cells transfected with siRPS4X-A and siRPS4X-D performed in duplicata). Data are the mean ± SE.

We next investigated the effect of RPS4X depletion on OVCAR-3 and SK-OV-3 cell growth. As indicated in Figure 4, two different siRNAs against RPS4X (hereafter designated siRPS4X-A and siRPS4X-D) significantly decreased the growth rate of OVCAR-3 and SK-OV-3 cells. We further analyzed the cell cycle of transfected cells by FACS analysis. As indicated in the summary histogram of Figure 4E, siRPS4X OVCAR-3 transfected with siRPS4X sequences showed an increase in S phase with a concomitant decrease in the G1 phase of the cell cycle compared to control siRNA transfected cells. Based on the growth rate (Figure 4C), these results suggest that the siRPS4X stalls OVCAR-3 cell proliferation in the S phase of the cell cycle. SK-OV-3 transfected with siRPS4X sequences exhibited an increase in the G2/M phase of the cell cycle with a concomitant decrease in the S phase. Based on the growth rate (Figure 4D), these results suggest that siRPS4X stalls SK-OV-3 cell proliferation in the G2/M phases of the cell cycle. Examples of FACS analyses are shown in the Additional file 3: Figure S2. The difference between RPS4X-depleted OVCAR-3 and SK-OV-3 cell cycle behavior is currently unknown. Nevertheless, siRPS4X decreased the proliferation rate in both cell lines.

**Figure 5:**
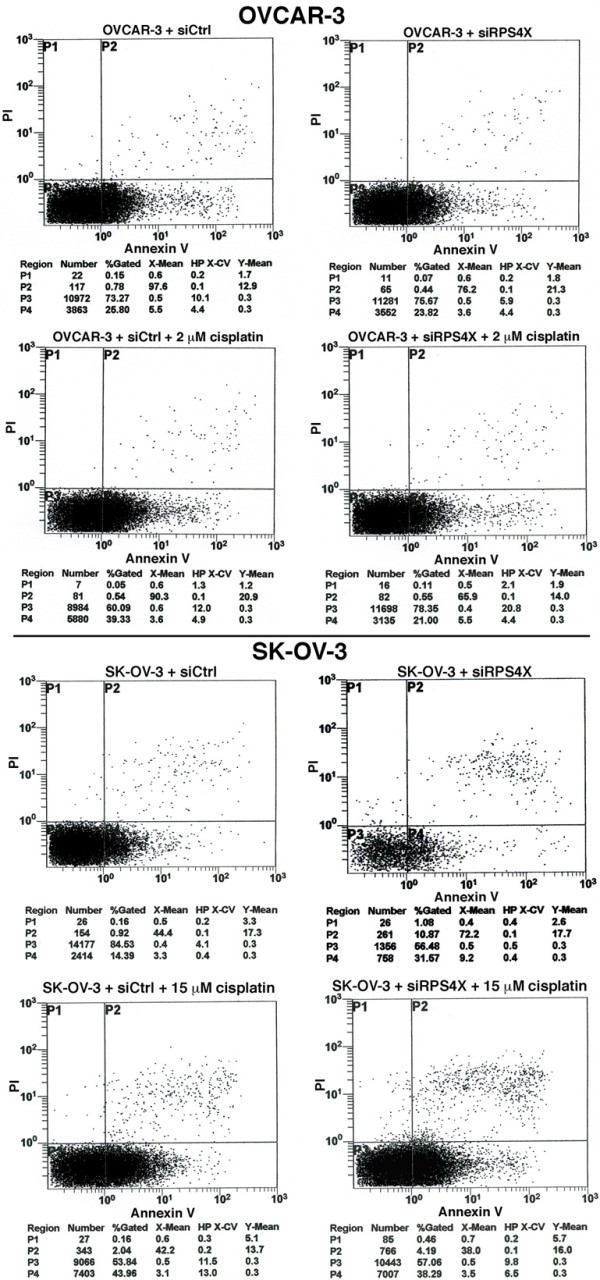
**Apoptotic and necrotic events in RPS4X-depleted cells were assessed in the presence of cisplatin by flow cytometry with Annexin V/PI staining.** Cells were transfected with the indicated siRNA sequences (siControl and siRPS4X-A) and 48 hours later cells were treated for 48 hours with the indicated concentration of cisplatin. In each graph, the P1 section (top left) represents necrotic cells, the P2 section (top right) represents both apoptotic and necrotic cells, the P3 section (bottom left) represents healthy cells, and the P4 section (bottom right) represents apoptotic cells. The number and percentage of cells in each section are indicated below each graph.

To determine whether a depletion of RPS4X had an impact on apoptosis, we analyzed siRPS4X transfected cells with a FITC-Annexin V assay and compared them to control siRNA transfected cells. A depletion of RPS4X protein in OVCAR-3 cells did not increase the percentage of apoptotic or necrotic cells in culture (Figure 5). In contrast, RPS4X depletion in SK-OV-3 cells increased apoptosis by 17% (Figure 5). These results indicate that the SK-OV-3 cells are more sensitive to the depletion of RPS4X protein than the OVCAR-3 cells.

### Depletion of RPS4X in OVCAR-3 and SK-OV-3 cells induces cisplatin resistance

**Figure 6:**
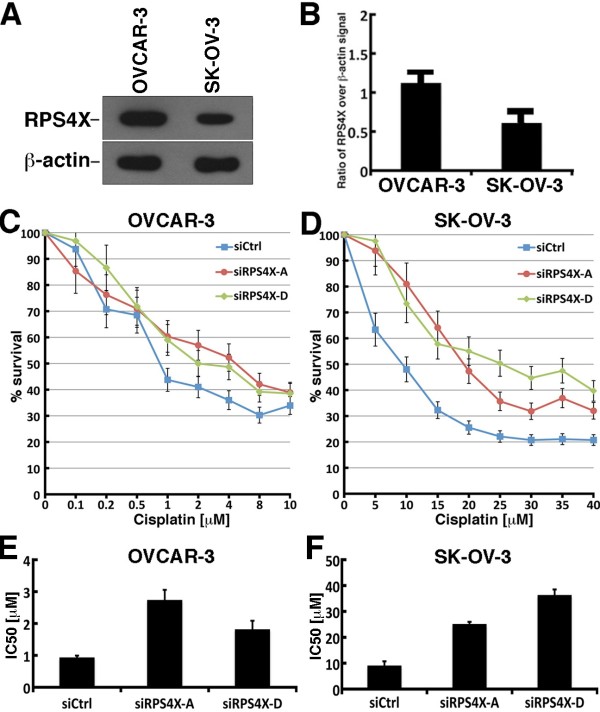
**RPS4X depletion increases cipslatin resistance in OVCAR-3 and SK-OV-3 cells. A**, One example of a western blot showing expression of RPS4X in untransfected OVCAR-3 and SK-OV-3 cells. β-actin is used as a loading control. **B**, Histogram presenting the ratio of RPS4X signal over β-actin signal from western blots. Experiments were performed in duplicate. **C**, Cisplatin dose response curves for transfected OVCAR-3 cells. **D**, Cisplatin dose response curves for transfected SK-OV-3 cells. Cells were transfected with the indicated siRNA molecules. Cisplatin dose response curves were determined by the sulforhodamine B colorimetric assay. **E**, Histogram representing the IC50 of OVCAR-3 transfected cells (from at least three transfections for each siRNA sequences) calculated from the drug response curves in A. Error bars represent the standard deviation. (Unpaired student *t-*test: *P* = 0.0084 for siRPS4X-A *vs* siCTRL and *P* = 0.025 for siRPS4X-D *vs* siCTRL). **F**, Histogram representing the IC50 of SK-OV-3 transfected cells (from at least three transfections for each siRNA sequences) calculated from the drug response curves in B. Error bars represent the standard deviation. (Unpaired student *t*-test: *P* = 0.00066 for siRPS4X-A *vs* siCTRL and *P* = 0.0001 for siRPS4X-D *vs* siCTRL). IC50 were estimated as described in materials and methods.

We first compared the expression of endogenous RPS4X in untransfected OVCAR-3 and SK-OV-3. As shown in Figure 6A and B, RPS4X protein levels were 1.5-fold higher in OVCAR-3 cells than SK-OV-3 cells. Although such cells were derived from patients with malignant ascites resistant to clinically relevant concentrations of cisplatin (http://www.atcc.org), we examined whether a depletion of RPS4X could increase cisplatin resistance further. As indicated in Figure 6C and D, RPS4X-depleted ovarian cancer cells were more resistant to cisplatin than control siRNA transfected cells. The calculated IC50 in OVCAR-3 cells for the control siRNA, siRPS4X-A, and siRPS4X-D were 0.9, 2.7, and 1.8 μM, respectively (Unpaired student *t*-test: *P* = 0.0084 for siRPS4X-A *vs* siCTRL and *P* = 0.025 for siRPS4X-D *vs* siCTRL) (Figure 6E). The calculated IC50 in SK-OV-3 cells for the control siRNA, siRPS4X-A, and siRPS4X-D were 9.1, 25.1, and 36.3 μM, respectively (Unpaired student *t*-test: *P* = 0.00066 for siRPS4X-A *vs* siCTRL and *P* = 0.0001 for siRPS4X-D *vs* siCTRL) (Figure 6F). These results indicate that cells that express low levels of RPS4X are more resistant to cisplatin and a depletion of RPS4X causes further cisplatin resistance in both serous epithelial ovarian cancer cell lines tested in this study.

We next analyzed the impact of cisplatin on cell death in transfected cells with a FITC-Annexin V assay. OVCAR-3 cells transfected with a control siRNA showed a 14% increase in apoptosis when treated 48 hours with 2 μM cisplatin (Figure 5). There was no significant increase in necrosis. In contrast, RPS4X-depleted OVCAR-3 cells did not exhibit an increase in apoptosis or necrosis after 48 hours of cisplatin treatment. Similarly, SK-OV-3 cells transfected with a control siRNA showed a 30% and 2% increase in apoptosis and necrosis respectively when treated for 48 hours with 15 μM cisplatin (Figure 5). In contrast, RPS4X-depleted SK-OV-3 cells showed only a 7% increase in apoptosis after 48 hours of cisplatin treatment (Figure 5). There was no increase in necrosis. Altogether these results indicate that RPS4X-depleted ovarian cancer cells are resistant to apoptosis induced by cisplatin.

### RPS4X interacts with YB-1 in ovarian cancer cells

We previously showed that RPS4X interacts with a tagged YB-1 in a breast cancer cell line [15]. To confirm this interaction in an ovarian cancer cell line, GFP-YB-1 and a control GFP expression vectors were transfected into SK-OV-3 cells. The next day the GFP-YB-1 construct was precipitated with an antibody against the GFP tag and the presence of RPS4X in the immunoprecipitate was detected by immunoblotting (see Additional file 4: Figure S3). Endogenous RPS4X was only found in the GFP-YB-1 immunoprecipitate indicating an interaction between RPS4X and YB-1 in ovarian cancer cells as well.

## Discussion

The expression of YB-1 in ovarian carcinomas has been correlated with a poor prognosis in several studies including one focused on serous ovarian cancer [7,23]. In contrast, there is one published report indicating no relationship between ovarian cancer patient survival and YB-1 expression [24]. Such contrasting results may be due to the small numbers of ovarian tumor samples, specifically of the serous type (less than 40 samples of both low and high grades), that were used in past studies [7,23,24]. Another confounding parameter in the interpretation of the results is the anti-YB-1 antibodies used in the different studies. Antibodies recognizing epitopes on the C-terminus [7,24] or the N-terminus portion of the YB-1 protein (our study) as well as the immunohistochemistry protocol can impact staining [25]. Finally, as our study focused exclusively on high-grade serous epithelial ovarian cancers, it is possible that within this subset of serous cancer YB-1 has little prognostic value. In contrast, the level of RPS4X may be a better prognostic biomarker than YB-1 in serous epithelial ovarian cancers.

Our recent analyses on YB-1 in breast cancer cell lines resistant to cisplatin have indicated an interaction between RPS4X and YB-1 [15]. As platinum-based regimen is a major treatment for ovarian cancer, we sought to determine whether the expression of RPS4X could have prognostic significance in this cancer type. In this study, we showed by immunohistochemistry that high expression of RPS4X correlated with overall survival and disease free progression. Low expression of RPS4X correlated significantly with tumor stage. These results suggest that RPS4X is a potential prognostic marker for high-grade serous epithelial ovarian cancer at the protein level. To our knowledge, there is no published study on RPS4X levels in ovarian cancers. RPS4X will need to be validated in an independent cohort of patients to confirm its clinical utility. In addition, a more quantitative way of measuring RPS4X expression, as for example real-time quantitative RT-PCR, could be envisioned.

An important aspect of RPS4X protein expression is its association with cisplatin resistance in different cell lines. The SK-OV-3 cell line is more resistant to cisplatin than the OVCAR-3 cell line [26,27]. Interestingly, the expression of endogenous RPS4X protein is lower in the more cisplatin resistant SK-OV-3 cell line than the OVCAR-3 cell line. In addition, a depletion of RPS4X in both the OVCAR-3 and SK-OV-3 ovarian cancer cell lines induced cisplatin resistance and is consistant with our previous data on RPS4X depleted breast cancer cell lines resistant to cisplatin [15]. Such results suggest that RPS4X would also have predictive values with regards to platinum-based chemotherapy. A major challenge with platinum-based regimen is that ovarian cancers can be either intrinsically resistant to treatments or will become resistant during therapy [5]. As the immunohistochemistry study was performed on serous high-grade ovarian tumors from patients who had not received chemotherapeutic treatment, the patients showing low expression of RPS4X in their tumor tissues at surgery could correlate with an intrinsic resistance to platinum-based drugs. More precisely, cancer cells with low expression of RPS4X present in high-grade tumors that have never been in contact with platinum would correspond to cells exhibiting a pre-existing mechanism for resistance to such a drug. The exact mechanism by which a depletion of RPS4X confers cisplatin resistance is not known. One hypothesis is that depletion of RPS4X could induce a ribosomal stress which in turn leads to a slower growth rate as observed in siRPS4X transfected ovarian cancer cell lines. It has been suggested that a reduced growth rate could constitute a significant event in the survival of cancer cells following a major stress like cisplatin treatment [28,29]. Finally, differential translation of not only several survival factors in addition to proteins critical in the control of apoptosis during cisplatin response may be affected as well in RPS4X-depleted cells. Large-scale proteomic analyses may help identifying such critical regulators in RPS4X-depleted cisplatin resistant cells. In addition, a thorough analysis of the impact of RPS4X levels on different types of reagents used in chemotherapy is also required.

## Conclusions

To conclude, we have established that RPS4X is a new promising prognostic marker for patients with high-grade serous ovarian cancer. More importantly, if RPS4X is shown to be predictive of cisplatin response either alone or in combination with other markers, this could be useful when selecting first line therapies for patients with serous ovarian cancer.

## Competing interests

The authors declare that they have no competing interests.

## Authors’ contributions

SPT, AMMM, and ML were responsible for the conception of the study. SPT and ML were responsible for the cell line studies. SPT, LM, LS, JM, ML were responsible for the assembly and analysis and interpretation of data. ML drafted the manuscript. LM and JM did the statistical analysis. DP provided study patients. LM and LS did the pathological assessment and interpretation of it. All authors contributed to revisions and approved the final version of the manuscript.

## Pre-publication history

The pre-publication history for this paper can be accessed here:

http://www.biomedcentral.com/1471-2407/13/303/prepub

## Supplementary Material

Additional file 1: Figure S1 Kaplan-Meier survival for low and high intensity of YB in high grade serous EOC. Click here for file

Additional file 2: Table S1 Wilcoxon-Mann–Whitney test for PRS4X expression (intensity) in EOC tissues and stage and residual disease of patients. Click here for file

Additional file 3: Figure S2 Examples of FACS analyses with the indicated cell lines and siRNA sequences. Click here for file

Additional file 4: Figure S3 Co-imunoprecipitation of endogenous RPS4X protein with GFP-YB-1 in transfected SK-OV-3 cells. Cells were transfected with GFP or GFP-YB-1 expression vectors and the next day GFP or GFP-YB-1 proteins were immunoprecipitated with an anti-GFP antibody. Endogenous RPS4X is co-immunoprecipitated only in cells transfected with the GFP-YB-1 construct. WCE = whole cell extract; anti-GFP = immunoprecipitation with an antibody against GFP. Bands corresponding to GFP-YB-1 and the endogenous YB-1 proteins are shown in the whole cell extract. Click here for file

## References

[B1] NIH Consensus Conference Ovarian cancer. Screening, treatment, and follow-up. NIH Consensus Development Panel on Ovarian Cancer JAMA 1995 273 6 491 497 10.1001/jama.1995.03520300065039 7837369

[B2] DiSaia PJ Bloss JD Treatment of ovarian cancer: new strategies Gynecol Oncol 2003 90 2 Pt 2 S24 S32 12928003 10.1016/s0090-8258(03)00341-x

[B3] Greenlee RT Hill-Harmon MB Murray T Thun M Cancer statistics, 2001 CA Cancer J Clin 2001 51 1 15 36 10.3322/canjclin.51.1.15 11577478

[B4] Marsh S Pharmacogenomics of taxane/platinum therapy in ovarian cancer Int J Gynecol Cancer 2009 19 Suppl 2 S30 S34 10.1111/IGC.0b013e3181c10513 19955911

[B5] Kartalou M Essigmann JM Mechanisms of resistance to cisplatin Mutat Res 2001 478 1–2 23 43 11406167 10.1016/s0027-5107(01)00141-5

[B6] Yahata H Kobayashi H Kamura T Amada S Hirakawa T Kohno K Kuwano M Nakano H Increased nuclear localization of transcription factor YB-1 in acquired cisplatin-resistant ovarian cancer J Cancer Res Clin Oncol 2002 128 11 621 626 10.1007/s00432-002-0386-6 12458343 PMC12164419

[B7] Oda Y Ohishi Y Basaki Y Kobayashi H Hirakawa T Wake N Ono M Nishio K Kuwano M Tsuneyoshi M Prognostic implications of the nuclear localization of Y-box-binding protein-1 and CXCR4 expression in ovarian cancer: their correlation with activated Akt, LRP/MVP and P-glycoprotein expression Cancer Sci 2007 98 7 1020 1026 10.1111/j.1349-7006.2007.00492.x 17459055 PMC11159905

[B8] Swamynathan SK Nambiar A Guntaka RV Role of single-stranded DNA regions and Y-box proteins in transcriptional regulation of viral and cellular genes FASEB J 1998 12 7 515 522 9576478 10.1096/fasebj.12.7.515

[B9] Stickeler E Fraser SD Honig A Chen AL Berget SM Cooper TA The RNA binding protein YB-1 binds A/C-rich exon enhancers and stimulates splicing of the CD44 alternative exon v4 EMBO J 2001 20 14 3821 3830 10.1093/emboj/20.14.3821 11447123 PMC125550

[B10] Ashizuka M Fukuda T Nakamura T Shirasuna K Iwai K Izumi H Kohno K Kuwano M Uchiumi T Novel translational control through an iron-responsive element by interaction of multifunctional protein YB-1 and IRP2 Mol Cell Biol 2002 22 18 6375 6383 10.1128/MCB.22.18.6375-6383.2002 12192037 PMC135634

[B11] Evdokimova V Ruzanov P Anglesio MS Sorokin AV Ovchinnikov LP Buckley J Triche TJ Sonenberg N Sorensen PH Akt-mediated YB-1 phosphorylation activates translation of silent mRNA species Mol Cell Biol 2006 26 1 277 292 10.1128/MCB.26.1.277-292.2006 16354698 PMC1317623

[B12] Ise T Nagatani G Imamura T Kato K Takano H Nomoto M Izumi H Ohmori H Okamoto T Ohga T Transcription factor Y-box binding protein 1 binds preferentially to cisplatin-modified DNA and interacts with proliferating cell nuclear antigen Cancer Res 1999 59 2 342 346 9927044

[B13] Pestryakov P Zharkov DO Grin I Fomina EE Kim ER Hamon L Eliseeva IA Petruseva IO Curmi PA Ovchinnikov LP Effect of the multifunctional proteins RPA, YB-1, and XPC repair factor on AP site cleavage by DNA glycosylase NEIL1 J Mol Recognit 2012 25 4 224 233 10.1002/jmr.2182 22434712

[B14] Guay D Garand C Reddy S Schmutte C Lebel M The human endonuclease III enzyme is a relevant target to potentiate cisplatin cytotoxicity in Y-box-binding protein-1 overexpressing tumor cells Cancer Sci 2008 99 4 762 769 10.1111/j.1349-7006.2008.00739.x 18307537 PMC11159512

[B15] Garand C Guay D Sereduk C Chow D Tsofack SP Langlois M Perreault E Yin HH Lebel M An integrative approach to identify YB-1-interacting proteins required for cisplatin resistance in MCF7 and MDA-MB-231 breast cancer cells Cancer Sci 2011 102 7 1410 1417 10.1111/j.1349-7006.2011.01948.x 21466612 PMC11159804

[B16] Heintz AP Odicino F Maisonneuve P Quinn MA Benedet JL Creasman WT Ngan HY Pecorelli S Beller U Carcinoma of the ovary. FIGO 26th Annual Report on the Results of Treatment in Gynecological Cancer Int J Gynaecol Obstet 2006 95 Suppl 1 S161 S192 17161157 10.1016/S0020-7292(06)60033-7

[B17] Rustin GJ Vergote I Eisenhauer E Pujade-Lauraine E Quinn M Thigpen T du Bois A Kristensen G Jakobsen A Sagae S Definitions for response and progression in ovarian cancer clinical trials incorporating RECIST 1.1 and CA 125 agreed by the Gynecological Cancer Intergroup (GCIG) Int J Gynecol Cancer 2011 21 2 419 423 10.1097/IGC.0b013e3182070f17 21270624

[B18] Le Page C Ouellet V Quinn MC Tonin PN Provencher DM Mes-Masson AM BTF4/BTNA3.2 and GCS as candidate mRNA prognostic markers in epithelial ovarian cancer Cancer Epidem Biomar 2008 17 4 913 920 10.1158/1055-9965.EPI-07-0692 18398031

[B19] Chatterjee M Rancso C Stuhmer T Eckstein N Andrulis M Gerecke C Lorentz H Royer HD Bargou RC The Y-box binding protein YB-1 is associated with progressive disease and mediates survival and drug resistance in multiple myeloma Blood 2008 111 7 3714 3722 10.1182/blood-2007-05-089151 18006704

[B20] Otsuka Y Kedersha NL Schoenberg DR Identification of a cytoplasmic complex that adds a cap onto 5′-monophosphate RNA Mol Cell Biol 2009 29 8 2155 2167 10.1128/MCB.01325-08 19223470 PMC2663312

[B21] Guay D Gaudreault I Massip L Lebel M Formation of a nuclear complex containing the p53 tumor suppressor, YB-1, and the Werner syndrome gene product in cells treated with UV light Int J Biochem Cell Biol 2006 38 8 1300 1313 10.1016/j.biocel.2006.01.008 16584908

[B22] Vichai V Kirtikara K Sulforhodamine B colorimetric assay for cytotoxicity screening Nat Protoc 2006 1 3 1112 1116 10.1038/nprot.2006.179 17406391

[B23] Kamura T Yahata H Amada S Ogawa S Sonoda T Kobayashi H Mitsumoto M Kohno K Kuwano M Nakano H Is nuclear expression of Y box-binding protein-1 a new prognostic factor in ovarian serous adenocarcinoma? Cancer 1999 85 11 2450 2454 10.1002/(SICI)1097-0142(19990601)85:11<2450::AID-CNCR21>3.0.CO;2-U 10357417

[B24] Huang X Ushijima K Komai K Takemoto Y Motoshima S Kamura T Kohno K Co-expression of Y box-binding protein-1 and P-glycoprotein as a prognostic marker for survival in epithelial ovarian cancer Gynecol Oncol 2004 93 2 287 291 10.1016/j.ygyno.2004.01.040 15099935

[B25] Woolley AG Algie M Samuel W Harfoot R Wiles A Hung NA Tan PH Hains P Valova VA Huschtscha L Prognostic association of YB-1 expression in breast cancers: a matter of antibody PLoS One 2011 6 6 e20603 10.1371/journal.pone.0020603 21695211 PMC3112203

[B26] Gibb RK Taylor DD Wan T O’Connor DM Doering DL Gercel-Taylor C Apoptosis as a measure of chemosensitivity to cisplatin and taxol therapy in ovarian cancer cell lines Gynecol Oncol 1997 65 1 13 22 10.1006/gyno.1997.4637 9103385

[B27] Egawa-Takata T Endo H Fujita M Ueda Y Miyatake T Okuyama H Yoshino K Kamiura S Enomoto T Kimura T Early reduction of glucose uptake after cisplatin treatment is a marker of cisplatin sensitivity in ovarian cancer Cancer Sci 2010 101 10 2171 2178 10.1111/j.1349-7006.2010.01670.x 20678156 PMC11158957

[B28] LaRue KE Khalil M Freyer JP Microenvironmental regulation of proliferation in multicellular spheroids is mediated through differential expression of cyclin-dependent kinase inhibitors Cancer Res 2004 64 5 1621 1631 10.1158/0008-5472.CAN-2902-2 14996720

[B29] Xing H Wang S Hu K Tao W Li J Gao Q Yang X Weng D Lu Y Ma D Effect of the cyclin-dependent kinases inhibitor p27 on resistance of ovarian cancer multicellular spheroids to anticancer chemotherapy J Cancer Res Clin Oncol 2005 131 8 511 519 10.1007/s00432-005-0677-9 15924242 PMC12161266

