# Supplementary figures and images for: Low expression of the X-linked ribosomal protein S4 in human serous epithelial ovarian cancer is associated with a poor prognosis
Source: BMC Cancer. 2013 Jun 22;13:303. doi: 10.1186/1471-2407-13-303 (PMC3708827; doi:10.1186/1471-2407-13-303)

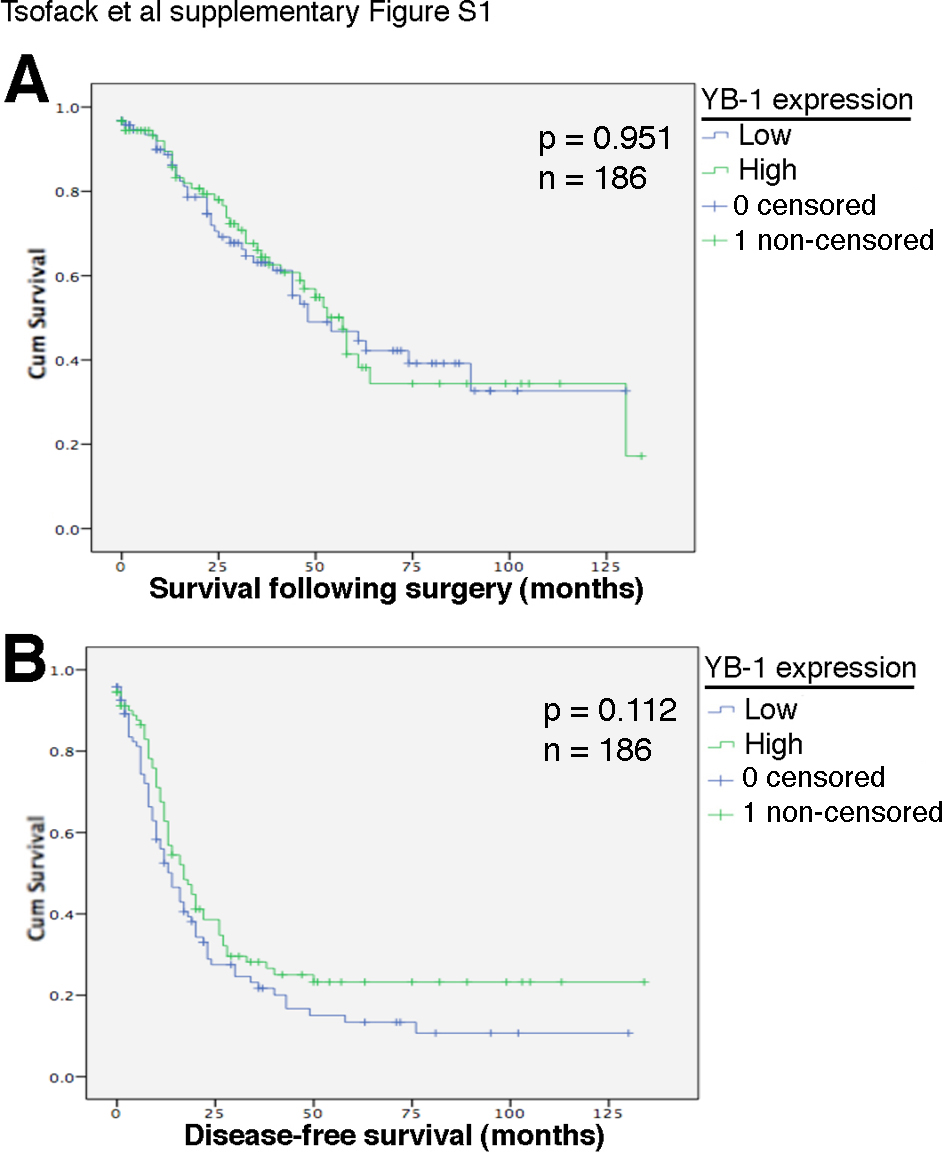

Supplement: Additional file 1: Figure S1 — Kaplan-Meier survival for low and high intensity of YB in high grade serous EOC. [file 1471-2407-13-303-S1.jpeg]

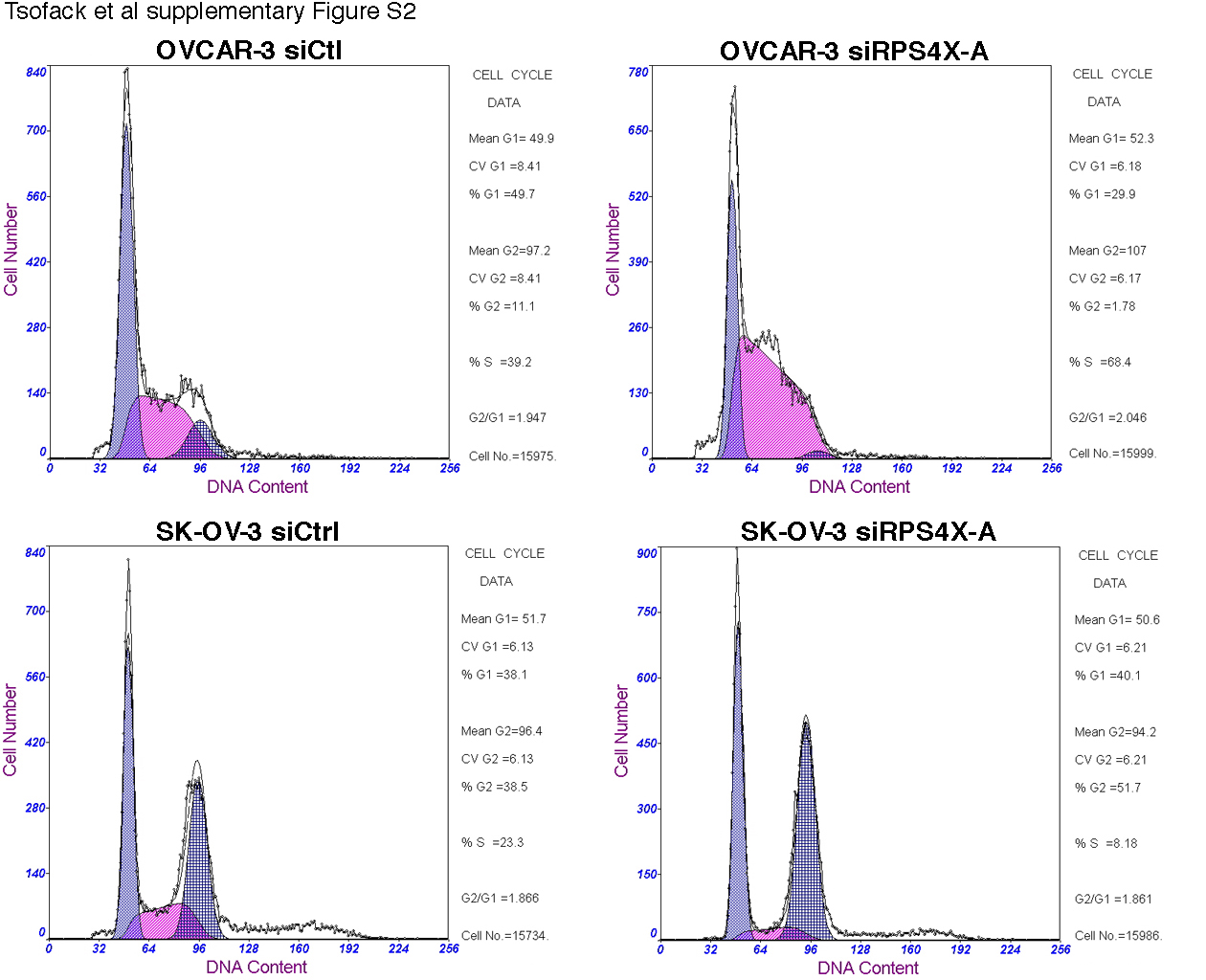

Supplement: Additional file 3: Figure S2 — Examples of FACS analyses with the indicated cell lines and siRNA sequences. [file 1471-2407-13-303-S3.jpeg]

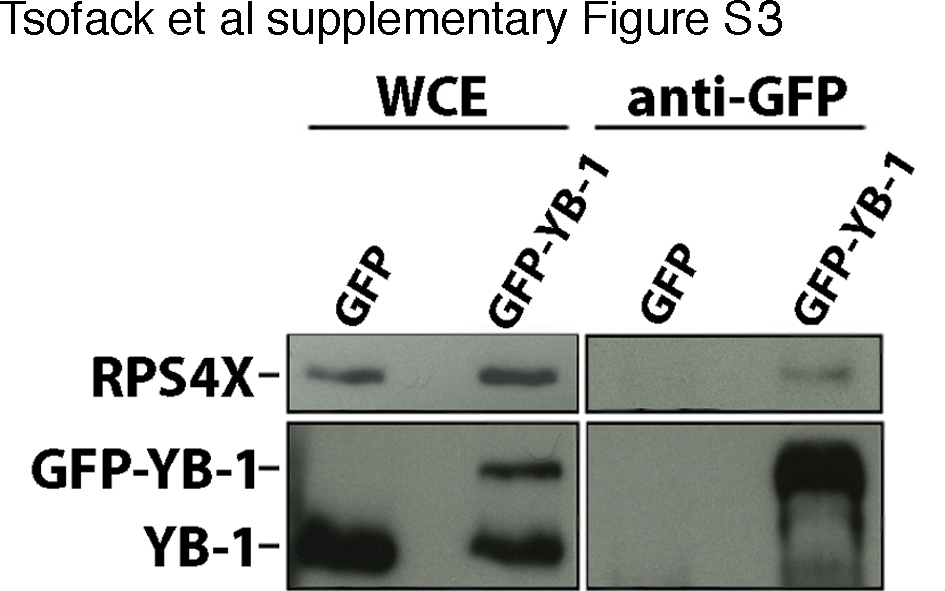

Supplement: Additional file 4: Figure S3 — Co-imunoprecipitation of endogenous RPS4X protein with GFP-YB-1 in transfected SK-OV-3 cells. Cells were transfected with GFP or GFP-YB-1 expression vectors and the next day GFP or GFP-YB-1 proteins were immunoprecipitated with an anti-GFP antibody. Endogenous RPS4X is co-immunoprecipitated only in cells transfected with the GFP-YB-1 construct. WCE = whole cell extract; anti-GFP = immunoprecipitation with an antibody against GFP. Bands corresponding to GFP-YB-1 and the endogenous YB-1 proteins are shown in the whole cell extract. [file 1471-2407-13-303-S4.jpeg]
