# Supplementary material for: Low expression of the X-linked ribosomal protein S4 in human serous epithelial ovarian cancer is associated with a poor prognosis
Source: BMC Cancer. 2013 Jun 22;13:303. doi: 10.1186/1471-2407-13-303 (PMC3708827; doi:10.1186/1471-2407-13-303)
Supplement: Additional file 2: Table S1 — Wilcoxon-Mann–Whitney test for PRS4X expression (intensity) in EOC tissues and stage and residual disease of patients. [file 1471-2407-13-303-S2.doc]

**Table S1. Wilcoxon-Mann-Whitney test for RPS4X expression (intensity) in EOC tissues and stage and residual disease of patients**

|  | Stage (1 vs 4) | Stage (1-2 vs 3-4) | Res. Dis. (0 vs >2cm) | Res. Dis. (0 vs all res. disease) |
| --- | --- | --- | --- | --- |
| Mann-Whitney U | 73,5 | 1882 | 581 | 1183 |
| Z | -2,028 | -2,109 | -1,929 | -1,458 |
| sig (2-tailed) | 0,043* | 0,032* | 0,054 | 0,145 |
| N | 36 | 190 | 88 | 141 |

* Correlation is significant at the 0.05 level (2-tailed).

Non-parametric Wicoxon-Mann-Whitney test were performed on clinical data and PRS4X staining intensity observed in the intra-epithelial area of ovarian tumors. Stage was evaluated according to the FIGO classification. “Sig.” in the table represents the p value from Pearson correlation test. N is the number of cases included in the statistical analysis.
